# Spatial Autocorrelation and Temporal Convergence of PM_2.5_ Concentrations in Chinese Cities

**DOI:** 10.3390/ijerph192113942

**Published:** 2022-10-27

**Authors:** Huan Wang, Zhenyu Chen, Pan Zhang

**Affiliations:** 1 School of Government and Public Affairs, Communication University of China, Beijing 100024, China; 2 School of Environmental Science and Engineering, Shanghai Jiao Tong University, Shanghai 200240, China; 3 School of International and Public Affairs, China Institute for Urban Governance, Shanghai Jiao Tong University, Shanghai 200030, China

**Keywords:** PM_2.5_, spatial correlation, temporal convergence, urban governance

## Abstract

Scientific study of the temporal and spatial distribution characteristics of haze is important for the governance of haze pollution and the formulation of environmental policies. This study used panel data of the concentrations of particulate matter sized < 2.5 μm (PM_2.5_) in 340 major cities from 1999 to 2016 to calculate the spatial distribution correlation by the spatial analysis method and test the temporal convergence of the urban PM_2.5_ concentration distribution using an econometric model. It found that the spatial autocorrelation of PM_2.5_ seemed positive, and this trend increased over time. The yearly concentrations of PM_2.5_ were converged, and the temporal convergence fluctuated under the influence of specific historical events and economic backgrounds. The spatial agglomeration effect of PM_2.5_ concentrations in adjacent areas weakened the temporal convergence of PM_2.5_ concentrations. This paper introduced policy implications for haze prevention and control.

## 1. Introduction

In 2012, China added PM_2.5_ to the newly revised Environmental Air Quality Standard and the Ministry of Environmental Protection (MEP) announced that 74 cities, mainly in major economic zones, would implement the new standards. As of 2018, according to the latest China’s Ecological Environment Bulletin released, 64.2% of cities at and above the prefectural level still had air quality that fell short of the national standard and haze or PM_2.5_ pollution was still severe. In addition, the air pollution discharged by China may diffuse or be transported to other countries through airflow, thereby causing more serious effects [1]. China’s haze governance is crucial to the cleanness of global air. Thus, given the natural characteristics of the fluid atmosphere, we aimed to determine the correlation of PM_2.5_ pollution between areas in China. With the deepening of haze governance in China [2], we also aimed to determine how the historical haze pollution patterns had changed over time. It is particularly necessary to discuss the above issues based on the haze pollution data in long time series because understanding the spatial and temporal distributions and evolutionary trends of haze pollution in different areas of China is the foundation for formulating prevention and control instruments for haze pollution.

Numerous studies were conducted on haze pollution in natural science, humanities, and social science [3,4,5,6,7,8,9]. The main research foci included its chemical composition [10,11,12], measurement technology [13,14], the formation mechanisms and influencing factors [15,16,17,18,19,20], haze pollution’s impact on the death rate [21,22], several diseases [23,24,25], and longevity [26,27]. However, literature on the geographical and temporal distributions of haze pollution most focused on a specific location [28,29,30,31] or a short period of time [32,33,34], and lacked systematic characterization based on long-time-series pollution data of national samples. In terms of research methods, the patterns of haze pollution distribution were mostly studied using simple descriptive analyses [18,28,29], and there were not enough detailed descriptions of the dynamic evolutionary characteristics of haze pollution distribution based on quantitative approaches like the convergence analysis and spatial correlation analysis.

Specifically, we systematically identified studies on the distributions of haze and divided them into the spatial distribution, temporal distribution, and integrated spatiotemporal distributions according to the different research angles, and then reviewed the progress of haze research from each perspective. First, most literature on the spatial distribution of haze centered on individual cities [28,30,35], a specific province (state) [29,36], or a specific urban cluster [32,37]. For example, Chen et al. collected and analyzed hourly PM_2.5_ data of Nanjing City area [28]. Yang and Christakos examined the daily PM_2.5_ level in Shandong Province in 2014 using Bayesian maximum entropy [36]. DeGaetano and Doherty analyzed the hourly PM_2.5_ concentrations from 20 stations in New York in 2003 and found a strong correlation among the stations [35]. Bai et al. examined the air quality situation in the Yangtze River Economic Zone of 2015 and identified influences of natural and urbanization factors [32]. Generally, the geographical distribution of PM_2.5_ was found to be related to the geographic location, seasonal variation, and meteorological factors such as temperature, humidity, and wind. [28,38,39,40].

Second, studies also used both single-year data to examine the temporal distribution of haze pollution [41,42] and multi-year data for analysis [43,44]. In the temporal dimension, seasonal variation was closely related to pollutant concentrations [42]. In China, PM_2.5_ concentrations showed seasonal variation [45]. For example, Zhang et al. collected meteorological bureau air pollutant data from 16 locations in different areas of China between 2006 and 2007 and found that the chemical composition of air pollution differed between urban and rural areas significantly and that most aerosol species reached maximum concentrations in winter [41]. Jin et al. examined 2015 data from 109 cities across China using the Bayesian spatiotemporal model and confirmed that daily pollutant concentrations peaked in winter [46]. Shi et al. further analyzed the nexus between changes in PM_2.5_ levels and Chinese urban morphology in 279 cities [42]. Gupta examined the monthly average PM_10_ and total suspended particulate concentrations in Delhi, Mumbai, Kolkata, and Chennai, from 1991 to 2003 using a time series analysis, and found that the trends of the PM_10_ indicators over the study period in the four cities were stable and positive [44]. Guo et al. analyzed the 2015 to 2017 air quality data from 366 Chinese cities and showed that the annual average pollution was reduced yearly [43].

Third, from the viewpoint of the integrated spatiotemporal haze distributions, most of the data was acquired from environmental monitoring stations [47,48,49], extrapolation of pollution emissions based on other variables or the inversion of satellite image data [50,51], and mobile Internet data [52], as well as other methods. Chow et al. studied air quality data from over 40 observation sites in Southern California in 1987 and revealed that maximum concentrations of PM_2.5_ and PM_10_ were reached in the fall at one-half to two-thirds of the sites. These pollutant levels were dominated by PM_2.5_ and related to the geographic location [47]. Xu et al. explored the temporal and geographical trends and socioeconomic factors using China’s air quality index data over long periods, finding an overall nationwide declining trend of air pollution, but a concentrated and increasing trend in North China [49]. Jin et al. found that China had seen spatially varying increases in PM_2.5_ emissions in the last ten years, with those in the eastern provinces increasing the most, and that provinces within the same region had a greater effect on each other [50]. Liu et al. retrieved aerosol optical depth data and estimated the PM_2.5_ concentration in combination with land use and meteorological data [51]. Yu et al. used the Google Maps application to obtain historical location data from smartphones to describe the extent of personal mobility and exposure to haze, and highlighted the importance of historical location data in relevant studies [52].

The above review shows that although studies were done on the spatial and temporal distributions of haze, there is still room for progress. First, the existing literature mostly focused on single cities or city clusters, with relatively few cross-regional studies. China is a vast country, and the analysis of only one economic zone cannot reflect the whole situation of China’s haze pollution. As a complete social economy, it is essential to holistically obtain the geographic spread of haze for the formulation of atmospheric environmental policies. Second, the temporal evolution of haze in the past was mostly analyzed by trend analysis, but no future predictions were made. Although trend analysis can illustrate the annual changes in haze, it cannot further describe the future trends of different haze levels. Convergence analysis can provide support for future evolutionary trends of haze. Furthermore, combining spatial distribution and temporal analyses can provide a more comprehensive panorama of changes in haze. Finally, from a data point of view, it was not until 2012 that PM_2.5_ was included in China’s MEP’s air quality standard. Owing to the lack of long-term data, previous studies either had a short time series or performed data estimation, which made it difficult to ensure completeness and accuracy. 

Therefore, in this study, data were collected from the global PM_2.5_ grid concentrations developed by an internationally renowned research team [53], spanning from 1998 to 2016. These data were used to illustrate the temporal convergence and spatial correlation of 340 major cities throughout China through spatial metric modeling, thereby presenting a comprehensive spatial and temporal distribution status of haze. The results of the paper also have important practical implications for the promotion of inter-regional collaborative control of urban haze.

## 2. Materials and Methods

### 2.1. Analysis Sample

Before 2012, there was no way to directly obtain environmental monitoring station observations for PM_2.5_ data in Chinese cities [18]. The data of this paper were aggregated from global PM_2.5_ grid concentration data developed by an internationally renowned research team, and they retrieved ground-level PM_2.5_ concentration values by integrating aerosol optical images from multiple satellites after removal of ground dust and sea salt [53]. Generally, each city was divided into several thousand or even tens of thousands of grids and PM_2.5_ concentration data of these grids were aggregated to the city level each year. These data have also been used by many studies to do academic research [54]. The analysis included 340 major cities of China covering almost all cities under the jurisdiction of provinces, municipalities, or autonomous regions in mainland China, which were highly representative. The database consisted of the yearly average PM_2.5_ levels in these 340 major cities between 1998 and 2016, and the national representativeness of the urban samples and the long time series of the data provided a good basis to study the spatial and temporal distribution features of PM_2.5_.

### 2.2. Analysis Methods

#### 2.2.1. Spatial Correlation Model

We used the global Moran’s I index to study whether the yearly average PM_2.5_ concentrations of the 340 cities were spatially correlated [55] according to the following steps: 

First, we determined the spatial distance weight matrix (*W*) between the 340 cities. When *i* = *j*, ${\tilde{\omega }}_{ij}$ took the value of 0; otherwise, ${\tilde{\omega }}_{ij}$ took the reciprocal of the spatial distance ($d_{ij}$) between cities *i* and *j*, as shown in Equation (1).

(1) $$W=\left(\begin{array}{ccc} {\tilde{\omega }}_{11} & \ldots & {\tilde{\omega }}_{1n}\\ \ldots & {\tilde{\omega }}_{ij} & \ldots \\ {\tilde{\omega }}_{n1} & \ldots & {\tilde{\omega }}_{nn} \end{array}\right);\;\mathrm{when}\;i=j,\;{\tilde{\omega }}_{ij}=0;\;\mathrm{when}\;i\neq \;j,\;{\tilde{\omega }}_{ij}=\frac{1}{d_{ij}}$$

Second, row standardization of the spatial weight matrix was conducted, i.e., each element of the matrix ${\tilde{\omega }}_{ij}$ was divided by the sum of its row elements to obtain a new matrix element $\omega_{ij}$. Then, the sum of each row element of such a standardized weight matrix is 1, as shown in Equation (2).

(2) $$\omega_{ij}=\frac{{\tilde{\omega }}_{ij}}{\sum_{j=1}^{n}{\tilde{\omega }}_{ij}}$$

Third, the global Moran’s I index of the annual average PM_2.5_ data of the 340 cities was calculated based on Equation (3). In Equation (3), $PM_{i}$ and $PM_{j}$ are the annual average PM_2.5_ data in the *i*th and *j*th cities, respectively, and $\bar{PM}$ is the average of the annual average data in the 340 cities.

(3) $$I=\frac{n}{\sum_{i=1}^{n}\sum_{j=1}^{n}\omega_{ij}}\times \frac{\sum_{i=1}^{n}\sum_{j=1}^{n}\omega_{ij}\left(PM_{i}-\bar{PM}\right)\left(PM_{j}-\bar{PM}\right)}{\sum_{i=1}^{n}{\left(PM_{i}-\bar{PM}\right)}^{2}}$$

In general, the range of values of the global Moran’s I is [–1, 1]. If global Moran’s I is positive, it means the existence of positive spatial autocorrelation, i.e., low-value areas are close to low-value areas, while high-value areas are close to high-value areas; if the value of global Moran’s I is negative, it means the existence of negative spatial autocorrelation; if the value of global Moran’s I equals zero, it means no spatial autocorrelation [55].

#### 2.2.2. Temporal Convergence Model

We used convergence analysis, which emerged from economics, to examine the temporal variation of the mean annual PM_2.5_ concentrations in various regions [56,57]. Using this approach, we could test if cities with higher starting PM_2.5_ concentrations would have more sharp declines in yearly mean PM_2.5_ concentrations than cities with lower initial ones. If so, this would imply that the yearly average PM_2.5_ concentrations would gradually converge with time. The general formula for the convergence analysis is shown in Equation (4):

(4) $$\frac{1}{T}ln\left(\frac{PM_{i,t}}{PM_{i,t-T}}\right)=\alpha +\lambda \overset{n}{\underset{j=1}{\sum }}\omega_{ij}ln\left(\frac{PM_{j,t}}{PM_{j,t-T}}\right)+\beta ln\left(PM_{i,t-T}\right)+\varepsilon_{i,t}$$

In Equation (4), $ln\left(\frac{PM_{i,t}}{PM_{i,t-T}}\right)$ denotes the natural logarithm of the ratio of the annual mean PM_2.5_ concentration in city *i* in year *t* to the annual mean PM_2.5_ concentration in that city in year *t − T*. *α* is the intercept term of the regression model, $\varepsilon_{i,t}$ represents the error term, and *λ* (lambda) is the spatial autocorrelation coefficient. When *λ* is significantly positive, it suggests a positive spatial correlation exists. When *λ* is significantly negative, it shows a negative spatial correlation exists. When *λ* is zero, it means the result of convergence analysis when no spatial correlation is considered. *β* is the regression coefficient for examining convergence. When *β* is significantly negative, it supports the existence of convergence.

## 3. Results

### 3.1. Spatial Correlation Analysis Results

We measured the global Moran’s I of the annual average PM_2.5_ concentrations of every city to examine the correlations between their spatial distributions. As Table 1 shows, the global Moran’s I values were all significantly positive. Thus, cities of high (low) yearly average PM_2.5_ levels were close to those of other cities of high (low) levels. According to the temporal evolution of the spatial correlation, the global Moran’s I tended to fluctuate and became larger, thereby indicating that the spatial correlation of China’s haze had tended to increase over time.

### 3.2. Temporal Convergence Analysis Results

We first examined the temporal convergence of the data using the ordinary least squares regression model (OLS) without taking the spatial correlation into account (see the OLS models). The spatial autoregression model (SAR) was used to further test the temporal convergence (see the SAR models). In Table 2, ln(PM_2.5_) is the core independent variable in both the OLS and SAR models, whereas the results of the ln(PM_2.5_) row in the table show the regression coefficients and standard errors of the variable ln(PM_2.5_). In the SAR model, the lambda row shows the spatial autoregressive coefficients and their standard errors. In the OLS and SAR models fitted based on the data of each year, the regression coefficients of the ln (PM_2.5_) values were significantly negative in 2000, 2001, 2002, 2008, 2011, and 2016, thereby indicating that the average annual PM_2.5_ concentrations were convergent over time. Meanwhile, the lambda coefficients in several SAR models were significantly positive, which further confirmed the positive spatial correlation.

We performed panel data convergence analysis to further test the temporal convergence characteristics in Table 3. Models 1–4 are the results of the ordinary panel data model analysis, whereas Models 5–8 are spatial autoregressive panel data model analysis results. For each model, we first performed a Hausman test and then selected either a fixed-effects model or a random-effects model. The strict standard errors are presented in brackets. Models 1 and 5 did not control for any fixed effects, Models 2 and 6 controlled for year fixed effects, Models 3 and 7 controlled for city fixed effects, and Models 4 and 8 controlled for both effects. In Models 1–4, the regression coefficients of ln(PM_2.5_) were all significantly negative (−0.426, *p* < 0.01; −0.691, *p* < 0.01; −0.426, *p* < 0.01; −0.6913, *p* < 0.01), thereby indicating the existence of temporal convergence. In Models 5–8, the *λ* coefficients of the models were all significantly positive (0.959, *p* < 0.01; 0.9817, *p* < 0.01; 0.9592, *p* < 0.01; 0.9817, *p* < 0.01), thereby indicating a positive spatial correlation in the annual mean PM_2.5_ concentrations. In Models 5–8, the regression coefficients for ln(PM_2.5_) also remained significantly negative (−0.2236, *p* < 0.01; −0.5679, *p* < 0.01; −0.2236, *p* < 0.01; −0.5679, *p* < 0.01), thereby further demonstrating the existence of temporal convergence of annual mean PM_2.5_ concentrations and our core findings were robust.

## 4. Discussion

The positive spatial correlation of the yearly average concentrations of PM_2.5_ in Chinese cities indicated a spatial agglomeration effect of haze pollution, i.e., high-pollution areas were clustered with high-pollution areas, while low-pollution areas were clustered with low-pollution areas. Moreover, the global Moran’s I index suggested a growing trend over time, which showed that the spatial autocorrelation of haze pollution was gradually increasing in various regions of China. Previous studies explored the spatially correlation features of China’s haze pollution and explained the reasons for the spatial correlation in the following aspects. First, there was strong spatial clustering in the economic structure of China’s regions [58], i.e., the economic institutions in neighboring regions were all relatively similar. Industry was a main source of haze in China, and the geographical clustering of industrial structures could lead to the spatial clustering of pollution [2,59]. Second, from the perspective of natural geographical factors, some adjacent municipal administrative areas located in the same basin or depression, the terrain feature of which was not conducive to the dissipation of pollutants and could easily form agglomeration phenomena [60]. Third, owing to the mobility of haze, when the pollution level of an area was high, the haze tended to radiate with mobile sources such as wind, transportation, and atmospheric circulation, which enhanced the spatial clustering effect of haze pollution [2,61].

The positive spatial correlation of haze pollution has important implications for China. Owing to the spatial agglomeration of haze pollution, the dependence of neighboring regions on economic development, and the spatial mobility of the atmosphere, haze abatement efforts are often difficult to achieve by a single local government. Therefore, there is a need to strengthen the communication and cooperation between neighboring highly polluted areas, ensure the sharing of pollution information, use consistent policy tools, conduct unified policy implementation, and coordinate governance means in order to avoid the transfer of pollution sources to neighboring areas and “free-riding” behaviors among neighboring local governments [62]. Furthermore, industrial restructuring and collaborative planning of the surrounding regions should be conducted by considering the resource endowment of each region and the topography, wind direction, humidity, and other natural conditions of overall planning to avoid the spread of haze pollution to neighboring regions and the introduction of industrial policy conflicts. Finally, when neighboring governments cooperate to deal with haze pollution, they should also strengthen inter-regional exchanges and endeavor to enhance the effectiveness of haze control by learning from each other’s experiences.

There was temporal convergence of PM_2.5_ in Chinese cities, i.e., haze pollution decreased faster in high-polluted areas than in low-polluted areas, which would eventually lead to a gradual convergence of haze pollution levels in different areas. This temporal convergence was inextricably linked not only to specific historical events, but also to China’s recent haze pollution control practices. Its temporal convergence was higher in 2008 than in other years; this might be related to the 2008 Beijing Olympic Games, which prompted Beijing to control haze pollution with neighboring provinces [63]. However, since 2013, when China suffered from widespread haze pollution, PM_2.5_ had received public attention and had gradually risen to the policy agenda [2,64]. The central government facilitated the reduction in PM_2.5_ concentrations in key areas of haze pollution by setting stricter PM_2.5_ concentration control targets for those areas, which helped to accelerate the control of haze in heavily polluted areas, thereby contributing to the convergence of haze [2].

The temporal convergence of haze in Chinese cities has important policy implications for improving the efficiency and equity of the management of haze pollution. For more polluted areas, the pollution can be reduced significantly with less efforts, whereas less polluted areas require greater efforts to achieve a similar treatment effect. Therefore, ensuring the convergence of haze pollution control can help to achieve national efficiency in environmental remediation. We can use the convergence result to consider how fair China is in controlling pollution. People in different regions have equal rights to clean air. From this perspective, ensuring the temporal convergence of haze pollution control can ensure that the air quality conditions in different regions eventually become homogeneous, thereby contributing to the achievement of inter-regional equity. The more polluted regions are the developing areas in western and central China, which rely on heavy industries, whereas the economies of the coastal areas, with particularly high levels of development, rely more on high-tech industries and services, and are less polluted. From the point of view of equity in economic development, each region should have the right to enjoy the benefits of development equally. However, more stringent pollution regulations can have a limiting and restrictive effect on the economic development of heavily polluted, underdeveloped regions [65]. Therefore, to ensure equity in economic development along with pollution regulation, the state should also give developing regions’ economies special consideration, especially the support of science and technology, capital investment and financing policies, and industry or fiscal transfers [66,67].

Although it is the first to comprehensively study the spatial correlation and temporal convergence characteristics of China’s haze pollution using long-time-series data from a national sample, the study has three limitations. First, there is a difference between absolute convergence and conditional convergence in the convergence analysis. No control variables need to be included when absolute convergence is examined, whereas control variables need to be included when conditional convergence is examined [56,57]. The purpose of this paper was to study the absolute convergence features of haze in various regions of China, so other economic and social development indicators were not included as control variables in the model. However, future studies can introduce relevant control variables to further investigate the conditional convergence characteristics. Second, the study mainly used historical data to uncover the spatial distribution and temporal convergence features of PM_2.5_ in China. The central government has implemented various initiatives to deal with haze pollution, including target management [2], and the practicality of the target setting of PM_2.5_ concentrations can be rethought by examining the convergence of future PM_2.5_ concentrations in each area. Third, this study just focused on PM_2.5_ concentrations and failed to analyze its chemical composition. Thus, future research can explore the evolution and variation of chemical components of PM_2.5_.

## 5. Conclusions

Spatial correlation and temporal convergence analyses were used to study the spatial and temporal distribution features of the average annual PM_2.5_ concentrations in various regions of China. The results showed that the global Moran’s I of the average annual PM_2.5_ concentrations in the cities was significantly positive in most years, thereby indicating significant positive spatial correlations in Chinese cities. In other words, cities with higher (lower) annual average PM_2.5_ concentrations were close to those with higher (lower) annual average PM_2.5_ concentrations. Furthermore, based on the cross-sectional data for each year, the results of the OLS models and SAR models indicated a convergent characteristic of the annual mean PM_2.5_ levels in Chinese cities in most years. The panel data results further confirmed the temporal convergence and positive spatial correlation features of PM_2.5_ in China, thereby confirming the robustness of the findings.

## Figures and Tables

**Table 1:** Global spatial correlation of PM_2.5_ concentrations.

**Year**	**1998**	**1999**	**2000**	**2001**	**2002**	**2003**
Moran’s I	0.208 ***	0.207 ***	0.232 ***	0.245 ***	0.248 ***	0.264 ***
**Year**	**2004**	**2005**	**2006**	**2007**	**2008**	**2009**
Moran’s I	0.242 ***	0.245 ***	0.241 ***	0.266 ***	0.255 ***	0.251 ***
**Year**	**2010**	**2011**	**2012**	**2013**	**2014**	**2015**
Moran’s I	0.251 ***	0.254 ***	0.246 ***	0.252 ***	0.250 ***	0.279 ***
**Year**	**2016**					
Moran’s I	0.267 ***					

Notes: *** *p* < 0.01. N = 340.

**Table 2:** Temporal convergence of PM_2.5_ concentrations in each year.

OLS Model	**Year**	**1999**	**2000**	**2001**
	ln(PM_2.5_)	0.0102 (0.0148)	−0.0885 *** (0.0217)	−0.1192 *** (0.0198)
	R^2^	0.0008	0.0489	0.1858
SAR Model	**Year**	**1999**	**2000**	**2001**
	ln(PM_2.5_)	0.0059 (0.0125)	−0.0527 *** (0.0179)	−0.2869 *** (0.0698)
	Lambda	2.803 *** (0.7285)	3.9296 *** (0.826)	−13.307 *** (4.985)
OLS Model	**Year**	**2002**	**2003**	**2004**
	ln(PM_2.5_)	−0.0211 ** (0.0101)	0.0217 (0.0142)	−0.0513 *** (0.0117)
	R^2^	0.009	0.0098	0.0262
SAR Model	**Year**	**2002**	**2003**	**2004**
	ln(PM_2.5_)	−0.0263 *** (0.0076)	−0.0033 (0.0159)	−0.0012 (0.0105)
	lambda	5.4189 ** (2.148)	2.175 *** (0.6496)	2.398 *** (0.217)
OLS Model	**Year**	**2005**	**2006**	**2007**
	ln(PM_2.5_)	0.0099 (0.0079)	0.0069 (0.0094)	0.0396 *** (0.0098)
	R^2^	0.0035	0.0016	0.0516
SAR Model	**Year**	**2005**	**2006**	**2007**
	ln(PM_2.5_)	−0.004 (0.0105)	−0.014 (0.0125)	−0.0214 ** (0.0097)
	lambda	5.8786 ** (2.4818)	8.618 *** (3.044)	3.068 *** (0.2558)
OLS Model	**Year**	**2008**	**2009**	**2010**
	ln(PM_2.5_)	−0.0997 *** (0.007)	0.0203 ** (0.0098)	−0.0065 (0.0094)
	R^2^	0.3227	0.0229	0.002
SAR Model	**Year**	**2008**	**2009**	**2010**
	ln(PM_2.5_)	−0.084 *** (0.014)	0.04 (0.0313)	0.0064 (0.008)
	lambda	0.6769 (0.4959)	−10.57 (15.84)	3.514 *** (0.6928)
OLS Model	**Year**	**2011**	**2012**	**2013**
	ln(PM_2.5_)	−0.0217 *** (0.0081)	−0.0144 (0.0098)	−0.0266 *** (0.0099)
	R^2^	0.0236	0.0105	0.0219
SAR Model	**Year**	**2011**	**2012**	**2013**
	ln(PM_2.5_)	−0.0256 ** (0.0109)	−0.0104 (0.0107)	−0.0066 (0.0104)
	lambda	12.744 *** (4.593)	1.257 (1.065)	2.62 *** (0.5838)
OLS Model	**Year**	**2014**	**2015**	**2016**
	ln(PM_2.5_)	−0.0041 (0.0088)	−0.0136 (0.0158)	−0.1174 *** (0.0126)
	R^2^	0.0007	0.0024	0.3226
SAR Model	**Year**	**2014**	**2015**	**2016**
	ln(PM_2.5_)	−0.0062 (0.0072)	−0.0408 *** (0.0112)	−0.0898 *** (0.0183)
	lambda	1.378 (0.909)	2.667 *** (0.153)	0.9019 ** (0.413)

Notes: *** *p* < 0.01, ** *p* < 0.05; N = 340; the robust standard error is presented in brackets.

**Table 3:** Temporal convergence based on sampled PM_2.5_ concentrations.

	**Model 1**	**Model 2**	**Model 3**	**Model 4**
ln(PM_2.5_)	−0.426 *** (0.0124)	−0.691 *** (0.0198)	−0.426 *** (0.0127)	−0.6913 *** (0.0204)
*λ*	/	/	/	/
Year fixed effects	/	Y	/	Y
City fixed effects	/	/	Y	Y
Hausman test	1496.93 ***	2070.43 ***	0	0
Model	fe	fe	re	re
R^2^	0.2669	0.4972	0.2731	0.5014
	**Model 5**	**Model 6**	**Model 7**	**Model 8**
ln(PM_2.5_)	−0.2236 *** (0.012)	−0.5679 *** (0.017)	−0.2236 *** (0.012)	−0.5679 *** (0.017)
*λ*	0.959 *** (0.0059)	0.9817 *** (0.001)	0.9592 *** (0.0059)	0.9817 *** (0.001)
Year fixed effects	/	Y	/	Y
City fixed effects	/	/	Y	Y
Hausman test	806.96 ***	3124.21 ***	0	−0.01
Model	fe	fe	re	re
R^2^	0.0675	0.0460	0.0677	0.0474

Notes: *** *p* < 0.01; N = 6120. In addition, fe denotes fixed-effects model, while re denotes random-effects model; Y denotes Yes, meaning the effects were controlled; *λ* denotes the spatial autocorrelation coefficient.

## Data Availability

The data are available upon request.
